# Supplementary material for: Inhibition of TNBC Cell Growth by Paroxetine: Induction of Apoptosis and Blockage of Autophagy Flux
Source: Cancers (Basel). 2024 Feb 22;16(5):885. doi: 10.3390/cancers16050885 (PMC10930888; doi:10.3390/cancers16050885)

Fig.2K. The effect of different concentration of PX on expression levels of apoptosis related protein in 4T1 and MDA-MB-231 cells

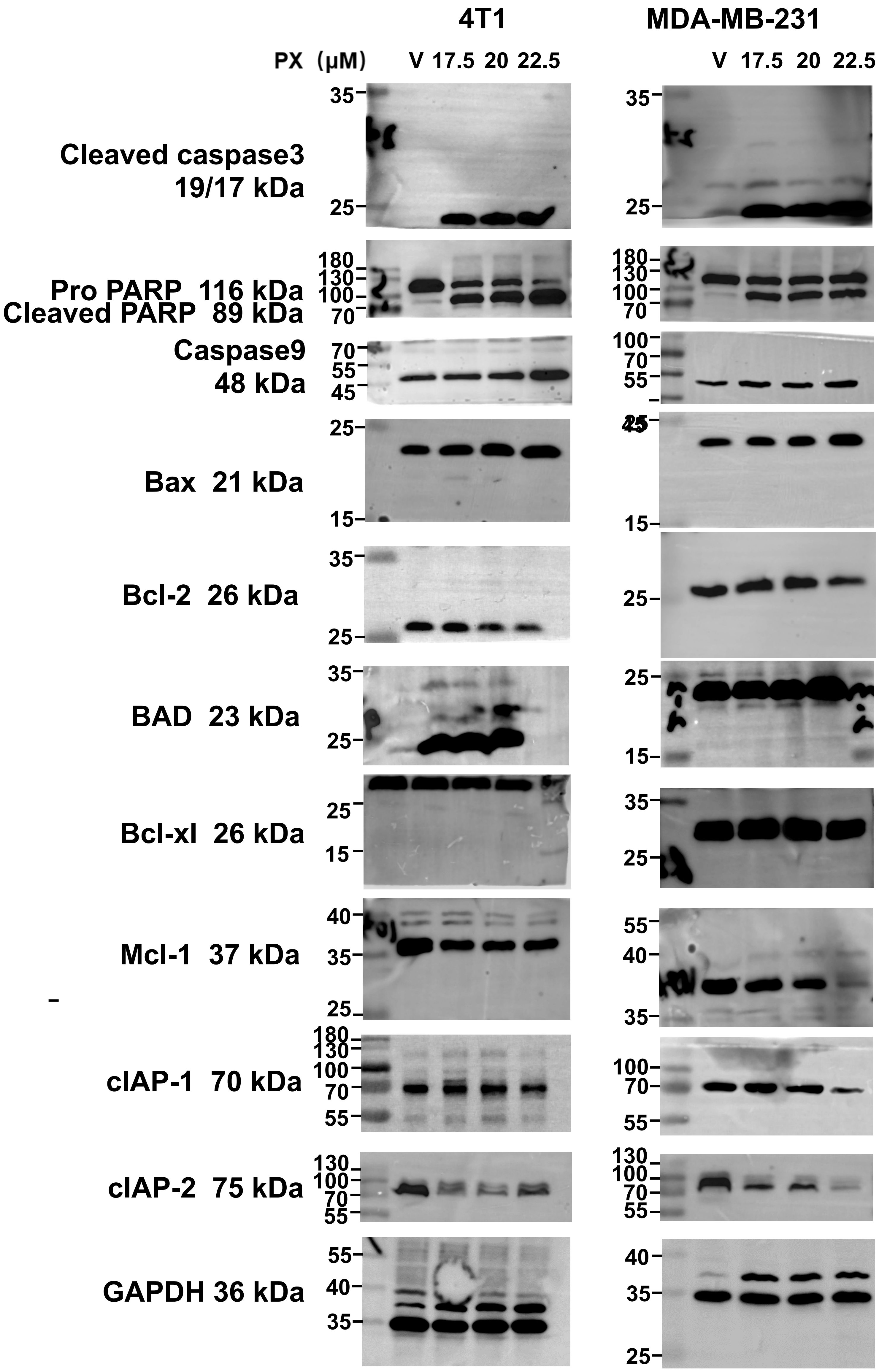

Fig. 3D. The effect of different concentration of PX on expression levels of autophapy related-protein AKT in 4T1, MDA-MB-231 cells

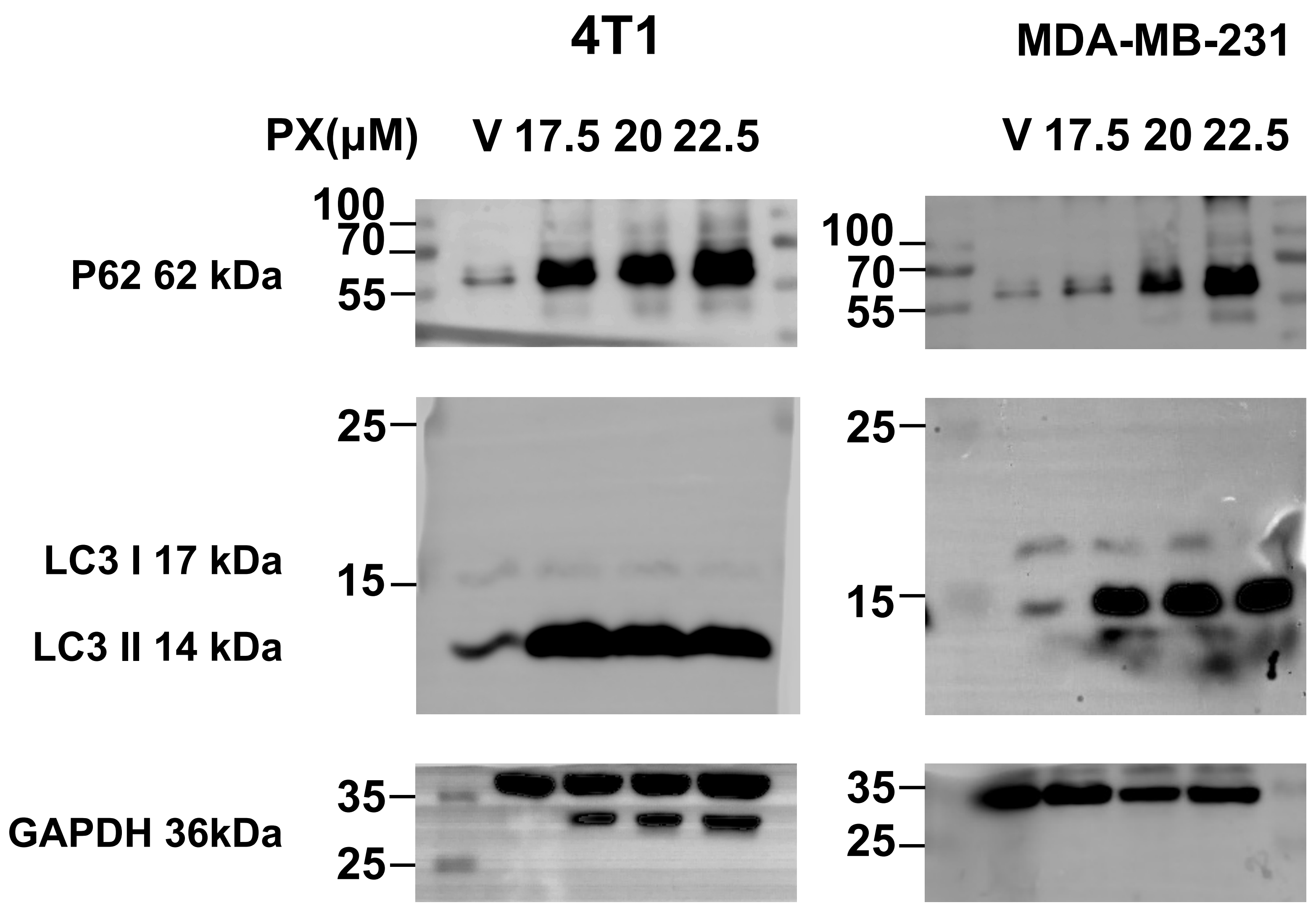

Fig. 4D. The effect of different concentration of PX on expression levels of pathway related-protein AKT in 4T1, MDA-MB-231 cells

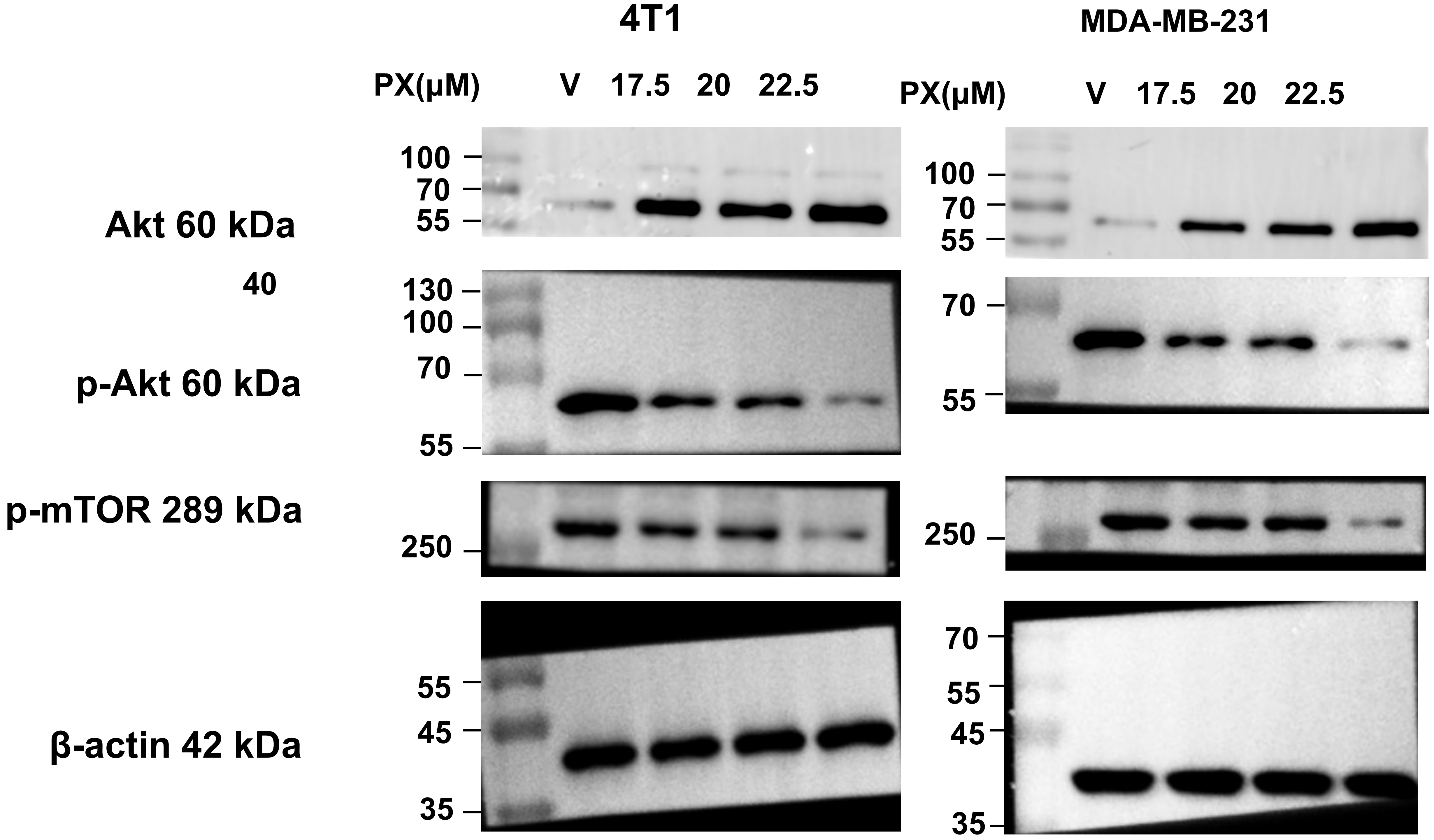

Supplement: Supplementary file 1 [file cancers-16-00885-s001.zip › cancers-2848766-supplementary.pdf]
